# Sustainable innovation in vector control requires strong partnerships with communities

**DOI:** 10.1371/journal.pntd.0007204

**Published:** 2019-04-25

**Authors:** Frederic Bartumeus, Guilherme B. Costa, Roger Eritja, Ann H. Kelly, Marceline Finda, Javier Lezaun, Fredros Okumu, M. Megan Quinlan, Delphine C. Thizy, Léa Paré Toé, Megan Vaughan

**Affiliations:** 1 CEAB-CSIC, Centre d’Estudis Avançats de Blanes, Girona, Spain; 2 CREAF, Centre de Recerca Ecològica i Aplicacions Forestals, Bellaterra, Spain; 3 ICREA, Institut Català de Recerca i Estudis Avançats, Barcelona, Spain; 4 World Mosquito Program, Fundação Oswaldo Cruz, Rio de Janeiro, Brazil; 5 Department of Global Health and Social Medicine, King’s College London, London, United Kingdom; 6 Ifakara Health Institute (IHI), Ifakara, Tanzania; 7 Institute for Science, Innovation and Society, University of Oxford, Oxford, United Kingdom; 8 Centre for Environmental Policy, Imperial College London, Ascot, United Kingdom; 9 Target Malaria, Imperial College London, London, United Kingdom; 10 Institut de Recherche en Science de la Santé, Bobo-Dioulasso, Burkina Faso; 11 Institute of Advanced Studies, University College London, London, United Kingdom; Northeastern University, UNITED STATES

Mosquito control is a multisectoral public service critical to advancing the 2030 sustainable development goals (SGDs). Inextricably linked to housing quality, urban planning, education, healthcare access, and other issues of economic equity and environmental justice, the challenge of mosquito-borne diseases can only be met by linking up diverse forms of expertise and advocacy [1].

Local communities are a key actor and crucial resource in this effort. As WHO’s 2017–2030 Global Vector Control Response reminds us, the engagement of affected communities is essential to building ‘sustainable control programmes that are resilient in the face of technical, operational, and financial challenges’ [2]. Though the form of participation will vary depending on the type of intervention, success hinges upon the degree to which citizens are effectively incorporated into the work of entomological research and mosquito abatement.

A range of innovations in vector control, however, are leading us to think more deeply about how local communities and other stakeholders participate in these endeavours. On the one hand, novel technologies of mosquito modification are expanding the range of disease control options. These technologies encompass diverse forms of genetic modification, including those making use of gene drive systems, and the introduction of inheritable alterations in the mosquito’s microbiome, as in the case of *Wolbachia*-infected strains. Depending on the particular trait incorporated into the mosquito, modified specimens can be used either to suppress the resident vector population or to replace it with a new strain that is refractory to transmission of the human pathogen. In all cases, the deployment of these technologies requires releasing laboratory-reared mosquitoes on an area-wide basis. As the history of large-scale mosquito releases suggests, the public legitimacy of this kind of intervention is always fragile—doubts about the purpose of the releases and misgivings about their long-term effects have in the past triggered intense local opposition [3]. Early attempts to define robust processes of governance for novel technologies of mosquito modification have emphasised the need to engage affected communities and other relevant stakeholders well in advance of deployment in disease-endemic areas and to develop models of communication, dialogue, and education attuned to local concerns and expectations [4–7].

In parallel to the development of new technologies of mosquito modification, we are also witnessing the emergence of innovative forms of citizen science specifically tailored to vector control challenges. Mobile communication technologies and digital platforms enlarge the potential pool of participants in mosquito surveillance efforts by enabling residents to gather and share entomologically relevant data [8]_._ These citizen science platforms create new channels of communication between mosquito control specialists and community members and can be an effective means of increasing the level of alert vis-à-vis endemic or emerging mosquito-borne diseases. Furthermore, these platforms also introduce new ways of quantifying the extent and intensity of public engagement with vector control interventions.

The confluence of innovations along these two fronts—new mosquito modification technologies and novel citizen science platforms—represents a unique opportunity to develop new approaches to public participation in the design, implementation, and evaluation of vector control programmes. A consultative workshop was held in Oxford in June 2018 to further this agenda [9]. Experts including entomologists, policy makers, development professionals, communication specialists, social scientists, and historians were asked to share experiences of success and failure in efforts to involve communities in mosquito control programmes past and present and to reflect on the challenges and opportunities that new technologies present. The meeting showcased the depth of practical experiences in engaging a wide spectrum of stakeholders. In what follows, we highlight key insights from our discussion and propose principles for robust community engagement with new vector control technologies.

The most immediate conclusion from our discussion was the need to have an expansive notion of ‘engagement’. Community participation in vector control is not simply a matter of obtaining individual or collective consent for a particular intervention but of integrating a diverse range of knowledge, experiences, and interests into the intervention itself. Mosquito control is notoriously labour intensive—the heterogeneity and dynamism of human–mosquito interaction requires local adaptation of tools and finely grained monitoring to ensure that disease control remains effective. Historically, community members have actively participated in the practical work of insect collection, insecticide application, or environmental modification [10–12]. Today, residents in intervention areas routinely act as project employees, advisers, and disseminators, offering indispensable technical advice, political support, and financial resources [13–15]. Novel technologies of vector control do not obviate the need for active involvement by local communities. On the contrary, they increase the significance of community authorisation and bring into sharper relief the importance of effective public dialogue, specifically when the importation and release of modified mosquitoes are concerned. ‘Engagement’ thus goes far beyond establishing one-way communication processes with local leaders or official stakeholders and requires developing a robust and context-specific strategy to involve citizens throughout the design, delivery, and evaluation of the intervention [16]_._

Second, our experience underscored the fluid nature of the relevant ‘community’ in vector control interventions. For one, the production, transportation, assessment, and release of laboratory-reared mosquitoes interconnect communities with varied backgrounds, expectations, and concerns, and located in diverse cultural, political, and regulatory contexts. Moreover, interventions that involve the release of self-sustaining mosquito populations are designed to operate at large ecological and temporal scales, casting doubt on hyperlocal understandings of the relevant community and on sporadic modes of participation. If the question of what constitutes the relevant community in a vector control effort is never self-evident, it is further complicated by the introduction of digital platforms for citizen-led data gathering and analysis. These tools expand the range of potential participants and create new forms of connectivity among residents in the area of intervention. One of the examples presented at our meeting was Mosquito Alert, a mobile-phone application that allows members of the public in Spain to send geo-referenced reports with images of mosquitoes or their breeding sites for elucidation by professional entomologists [17]. With almost 15,000 reports lodged since its launch in 2014, the application has served to monitor the spread of *Aedes albopictus* mosquitoes across Spain and was recently the source of the first-ever confirmed observation of *A*. *japonicus* in the country. In addition to serving as a tool of entomological surveillance, Mosquito Alert has also proved its value as a means of promoting social awareness and education about mosquito-borne diseases [18]_._ Another example discussed at the workshop was the recruitment and training of local residents in the Kilombero Valley, southern Tanzania, to locate swarms of *Anopheles* mosquitoes. Equipped with mobile phones, these volunteers report any sighting to entomologists at the Ifakara Health Institute, who then visit the site to verify, geo-locate, and sample the mosquito swarm [19]_._ The project builds on previous training of local residents in the identification of areas of high mosquito density with the help of a participatory global information system approach [20]. Whether they involve minimal training and target a large population of potential volunteers, as in the case of Mosquito Alert, or require the acquisition of new skills and their application to a strategic site of intervention, as in the Ifakara swarm searching project, these and similar experiences suggest that innovations in citizen science can effectively enhance mosquito surveillance and, in the process, create new channels of communication between experts and residents in affected areas. The ultimate result is a more active public vis-à-vis the relevant vector control challenge.

An expanded notion of engagement and greater attention to the community-making dimensions of vector control lead to an enriched understanding of participation, beyond the legally defined and restricted process of public consultation [21]. Recent experiences presented at the workshop, such as the experimental releases of *Wolbachia*-infected *Aedes* mosquitoes in Brazil [22][23], part of the World Mosquito Program, or the scoping research currently underway in several sub-Saharan countries for the possible deployment of modified *Anopheles* mosquitoes by Target Malaria [24], demonstrate the importance of continual, transparent, and open dialogue. Both projects have made a substantial investment of time and financial resources in building proactive communication and community engagement strategies. And both have adapted these strategies as the intervention evolved, piloting innovations in public outreach. The World Mosquito Program, for instance, has experimented with the establishment of ‘community reference groups’ to liaise with residents in the neighbourhoods and towns where the releases have taken place [25,26]. One key lesson from these ongoing processes of social innovation is that researchers, project managers, and funders cannot identify in advance the most appropriate strategy for articulating the multiple and often conflicting interests at stake, and must instead devise adaptable participation models based on a set of clear commitments. Similarly, the effective use of digital citizen science platforms requires flexibility in communication and data collection strategies in order to enable adaptation and responsiveness to specific goals (whether local or global) and to the capabilities and constraints of the relevant community, with the ultimate goal of ensuring codevelopment of the platform with its intended users. Each mosquito control project must thus develop its own participatory practice, building it over a period of time and evaluating it in constant dialogue with the communities hosting these interventions.

Finally, we would like to restate the importance of supporting independent social scientific research into the conditions for effective community participation in mosquito control [27,28]. Social scientists and experts in stakeholder engagement play a key role in the success of many individual programmes. Yet it is crucial to create platforms in which the experiences of different projects can be shared, contrasted, and disseminated while respecting confidentiality of individuals and communities. Meetings like the one we held in Oxford allow mutual learning between projects tackling comparable challenges under very different social and political conditions. They also encourage reflection on the longer historical trajectory of community participation in vector control. At a time when new technologies are reshaping the very meaning of appropriate community engagement, this sort of collective, comparative reflection becomes essential.

In sum, we need models of responsible innovation and tools for public engagement that are commensurate to the challenges and opportunities implicit in novel technologies of vector control. What constitutes the most relevant ‘community’ changes with the advent of new forms of mosquito modification, whose potential risks and benefits go well beyond locally circumscribed territorial units and, in some cases, will persist long after their initial deployment. Traditional forms and mechanisms of public participation are changing, as larger sections of the population can take part in mosquito surveillance and control activities with the help of new communication and data-sharing platforms. Independent, comparative research into the conditions that enable meaningful public engagement must play an important role in building the strong partnerships with communities and local institutions that are vital to achieving sustainable control of mosquito-borne diseases.
